# The College of Nursing (Limited by Guarantee)

**Published:** 1920-12-18

**Authors:** 


### The College of Nursing

### (Limited by Guarantee)

EDINBURGH CENTRE.

A meeting of members, convened for the purpose of considering the position of nurses under the Unemployment Insurance Act, was held in the Nurses' Club, 8 Drumsheugh Gardens, on Saturday, December 4. A discussion on the subject was opened by Miss May Dunbar, and the following resolution passed: "That this meeting resolves to request the College of Nursing to press for complete exclusion of nurses from the operations of the Act, on the ground that their rate of unemployment is so small as to be negligible; and, further, in the event of nurses not being so excluded, that the College of Nursing, in co-operation with other organisations representing nurses, be requested to take the necessary steps to set up a special scheme for nurses under Section 8 of the Act, and in this way contract out of the general scheme."

